# Annotations

**Published:** 1894-06-16

**Authors:** 


### ANNOTATIONS.

### Old-Fashioned Practice.

It is a common complaint against busy practitioners who have passed middle life that they tend to become very stereotyped and "old-fashioned" in their methods of practice. As a matter of fact, however, there ought not to be, and there need not be, any such thing as antiquated practice in medicine. It is true the average doctor can never by any possibility read all that is written. But the intelligent practitioner, whatever his age, has a way of keeping himself actively abreast of the times both in the science and the art of his calling. He has learnt the secret of selection in his studies. He knows what to read and what to let severely alone. The principles of selection for practical medical reading are very obvious. A man must maintain his knowledge of the parts and relations of the bodily organs fresh and bright; he must enlarge, and correct and improve, and make definite, his knowledge of human and comparative physiology; he must acquaint himself with the physiological action of remedies; he must be a pathologist; he must keep an eye upon new methods and details. All this he can easily do whilst carrying on a busy practice; because, he is not merely reading, he is seeing and handling every day the facts which constitute anatomy and physiology; he becomes a pathologist whether he will or not, merely by the daily honest and intelligent pursuit of his business; he is an experimental therapeutist on his own account. In short, the man who is honest, thorough, and intelligent at the bedside, and who knows how to read with judicious selectiveness, can never become old-fashioned, whatever his age. On the contrary, not only is he abreast of the times, but he becomes capable of giving intelligent and competent leadership to the times, and that by virtue of his age, accumulated knowledge and experience. In medicine as in other things, it is the man who makes the doctor, not the doctor who makes the man.

### Should Doctors Trade?

A good deal of surprise has been expressed at the announcement of the Incorporated Medical Practitioners' Association that it is prepared to recommend fire insurance offices and to receive commissions thereupon. That association also announces some other forms of commercial enterprise which, though less startling, are decidedly novel. Nothing of a novel kind in the way of commercial development startles us very much; but, so far as the medical profession is concerned, we have been accustomed to think that though all the world should rush to frenzy in search of the indispensable wherewithal to live, yet medicine would remain as staunchly true to the ways of a sober and honest dignity of action as the needle remains true to the pole. Frankly, is there any reason why the doctor should not act as a fire insurance agent, or why he should not sell tea or tripe? There appears to be no valid ethical reason why we should not keep open minds and turn our attention to any honest calling which we may have a latent capacity for. But still—but still, there may be objections, even though they are not of an ethical kind. There is such a thing as jumping out of the frying pan into a more actively uncomfortable place. We cannot help thinking that the combination of physic with fire insurance or tea agencies will diminish the profits from practice in a much higher ratio than it will produce profits of its own. Besides, will not the proper insurance agent or tea vendor be entitled to say of medical men who invade his province that from his point of view they are mere laymen and unqualified practitioners? The doctor, we admit, must do his best to live, but we do not think it will work at all to attempt to combine physic with commercial activity in the way put before us by the programme of the Incorporated Practitioners' Association. On the contrary, a loss of money, we are sure, will accompany the loss of professional dignity, and the doctor who is half starved by following his profession alone will be very likely to find himself starved entirely by combining medical practice with unlicensed poaching in other men's commercial preserves.

### The Prevention of Suicide.

Some few weeks ago we made an attempt to stem the rising tide of suicide. A writer in a provincial newspaper, so far from thanking us, offers sundry arguments to his public in favour of self-destruction. We need not remind classical scholars that this controversy is practically as old as civilisation. It surprises us a little, however, that an Englishman should revive it, and stand firmly by the old-world arguments. Suicide cannot be seriously held to be a desirable thing. At best it is an escape from unhappiness which is felt to be intolerable. A "hard lot" is frequently the incentive to self-destruction. It seems to be a circumstance as fixed as destiny that many persons shall experience a "hard lot." Well, but if the hard lot cannot be prevented, then it would be a gain if some means could be found of enabling men and women to stand up under it; to stand up, to endure it, to conquer it, and to have brave and free minds in spite of it. Following the *Spectator*, we maintained the thesis that the decay of religious faith coincides with an increase of suicides; and we pointed out that in Scotland, where religious faith has decayed but little, suicides are relatively infrequent; and that in the Salvation Army, where religious faith is exceedingly robust, suicide is practically unknown. Whereupon our critic retorts that the Salvation Army consists mostly of "idiots," and that we ourselves are so weak-minded as to be "pious." But really, if we grant that the Salvation Army consists mainly of "idiots," and that we ourselves are so imbecile as to feel that even "piety" may have its advantages, we fail to see how these things touch the controversy. This seems to us to be the whole question in a nutshell: The individual doctor tries to cure his patient, and he chooses the best means, whatever they may be, for his purpose. Doctors, in the mass, have an eye upon public material ills, and so far as they can they strive to mitigate, or to remove, or to prevent them. Now if a doctor perceives that religious faith, by steadying the mind and filling it with hope, mitigates many of the material ills of life, and where it is strong prevents some of them such as suicide, altogether, he will not be a wise doctor, he will not be an honest man, if he does not advise the remedy which he finds most potent for his purpose. The philosophic doctor regards all things philosophically, and when he finds religion medically serviceable, he recommends its use exactly as he would recommend any other remedy. Religion is undoubtedly of use to unhappy persons; therefore let all the unhappy by all means seek its consolations and its staying powers.

